# A comparison of rural Australian First Nations and Non-First Nations survey responses to COVID-19 risks and impacts: implications for health communications

**DOI:** 10.1186/s12889-022-13643-6

**Published:** 2022-06-30

**Authors:** Julaine Allan, Jodie Kleinschafer, Teesta Saksena, Azizur Rahman, Jayne Lawrence, Mark Lock

**Affiliations:** 1 University of Wollongong, School of Health and Society, Wollongong, NSW Australia; 2 Charles Sturt University, Bathurst, NSW Australia; 3 Western NSW Local Health District, Bathurst, NSW Australia; 4 University of Technology, Sydney, NSW Australia

**Keywords:** First Nations Australians, rural health, COVID-19, health risk communication

## Abstract

**Introduction:**

This study investigated differences between rural Australian First Nations and non-First Nations survey respondents’ perceptions of COVID-19-related risks and analysed other variables that could predict an exacerbation of anxiety related to COVID-19 harms.

**Methods:**

A cross-sectional online and paper survey of rural residents from the western regions of NSW, Australia, was conducted. Descriptive and multivariate statistical analyses were used to assess links between First Nations status and demographic measures including postcode, age, gender, education, rural or town/village location, proximity to medical services and living situation. The analysis included five items related to perceptions about COVID-19: perceived likelihood of contracting COVID-19 in the next 12 months, perceived harmfulness of the virus, how often people felt afraid, perception about respondents’ ability to do something about the virus and perceived economic impacts of the pandemic.

**Results:**

There were significant differences between First Nations (*n*=60) and non-First Nations (*n*= 639) respondents across all sociodemographic categories. The results reflect a significantly higher level of anxiety among the First Nations Australians in the sample: they felt afraid more often, felt it was highly likely they would catch the virus and if they did catch the virus perceived that it would be very harmful. Living with children under eighteen years of age and in small rural towns were key factors linked to feeling afraid of COVID-19 and First Nations status.

**Conclusion:**

Health risk communication in pandemic response should include an equitable focus on rural areas, recognising that First Nations Australians are a significant proportion of the rural population with different risk factors and concerns than those of non-First Nations Australians. This principle of First Nations-led design is critical to all health policy and planning. The Australian Government should include rural areas in planning pandemic responses, recognising that First Nations populations are a significant proportion of the rural population creating syndemic conditions.

**Supplementary Information:**

The online version contains supplementary material available at 10.1186/s12889-022-13643-6.

## Introduction

Globally, rural populations have experienced significant impacts from COVID-19. While infections spread more rapidly in highly populated areas, once the virus arrived in rural areas, mortality rates were higher, and economic and social impacts were more serious [1, 2]. Impacts on rural communities were attributed to generally poorer health, including chronic conditions, an older population, lower education, and employment that had to be undertaken in person [3].

Australia has reported low numbers of COVID-19 infections compared to many places in the world. Geographic isolation and good infection control have resulted in a small proportion of the population being affected by the disease [4]. At the beginning of the pandemic with limited experience of similar health crises, the government scrambled to identify and protect the country’s most vulnerable groups, including people with chronic illnesses, those in aged care and First Nations Australians. The phrase ‘First Nations Peoples’ refers to Indigenous peoples worldwide but especially in colonised nations of Australia, Aotearoa/New Zealand, Canada, and the United States. In this paper, the phrase ‘First Nations Australians and First Nations’ refers to the Aboriginal and Torres Strait Islander peoples in Australia [5]. First Nations Australians were found to be at greater risk of morbidity and mortality during past influenza pandemics [6], and gaps had been identified in existing disaster response plans, including a lack of information targeted to First Nations Australians [7].

People in rural and remote Australia were not specifically included as a vulnerable group in COVID response plans even though they were likely more at risk from COVID-19 for the same reasons as rural populations in other countries. First Nations Australians are a higher proportion of the population in rural areas compared to urban areas, and due to widespread racism, structural disadvantage and dispossession of land are disproportionately affected by poor physical and mental health, lower incomes and crowded housing prior to COVID-19 compared to non-First Nations Australians [8, 9]. COVID-19, converging with existing social and health conditions, including an epidemic of poor mental health, has the potential to cause a syndemic for First Nations Australians [9, 10]. Limited access to rural health care was further reduced during the pandemic due to service closures and fear of disease contagion. A move to virtual healthcare delivery was constrained in many rural areas because of unreliable connections and poor coverage [11]. This has been a more significant impact of COVID-19 on First Nations Australians who already had poorer access to digital devices and outcomes from virtual healthcare [12, 13].

While a NSW pandemic preparedness guideline released in July 2019 was based on extensive consultation with First Nations stakeholders, there was a notable absence of empirical research informing the strategy [14]. In addition, there was an absence of research examining rural First Nations Australian’s perceptions of COVID-19 risks and information and communication needs to better inform culturally safe community management and COVID recovery plans [15, 16].

Education level is a key factor in accessing and interpreting pandemic information and should be considered in the development of health communications. However, few studies examine the education level of respondents when assessing risk perceptions about COVID-19 [17, 18]. Studies of the current COVID-19 pandemic and previous Swine flu pandemic have found that health messaging has been confusing and difficult to understand, with written materials requiring above average reading ability and with limited attention paid to providing targeted messages to marginalized communities [18, 19]. However, it is not just education levels that are a factor in understanding pandemic information and responses. All infectious diseases disproportionately impact poor populations, women and First Nations peoples who experience health inequality because of these demographic characteristics [19]. Different consumer populations require nuanced communications that address their cultural milieu, including for First Nations Australians, a distrust of government and poor health care experiences that reduce access to healthcare and also acceptance of health communications [20, 21].

Risk perception and resultant behaviour are strongly influenced by personal, social and cultural contexts [22]. Two Australian studies have identified differences between First Nations and non-First Nations respondents in relation to COVID-19. One study found that non-Caucasian people were more likely to engage in protective health behaviours and included First Nations Australians in that group [23]. The other study found that the First Nations Australians perceived a greater risk from people who were not vaccinated [24]. However, there has been no investigation of the COVID-19 risk perceptions of First Nations Australians living in rural areas compared to those of non-First Nations rural populations. In particular, there is an absence of research examining rural First Nations people’s concerns about COVID-19 and its likely impacts to describe and compare factors that could better inform culturally safe communication strategies [15, 6]. To address this gap, this study investigated differences between rural-dwelling First Nations and non-First Nations survey respondents’ perceptions of COVID-19-related risks during the first COVID-19 lockdown in Australia and analysed other variables that could predict an exacerbation of anxiety related to COVID-19 harms.

## Methods

A cross-sectional online and paper survey of rural residents from the western regions of NSW was conducted.

The research team included two First Nations researchers (a Wiradjuri Woman and Ngiyampaa Man). A First Nations Reference Group was convened with members of the western NSW community and met monthly for the life of the project. This group oversaw the cultural safety and sensitivity of the project [25]. For example, they reviewed and amended the survey (e.g., changing problematic language and including additional socio-demographic questions about household types, living arrangements, the role of community leaders and the types of health services available). They also recommended data collection methods and advised on the implications of the results.

### Setting

Western NSW accounts for 29% of the NSW population and has the largest population of First Nations Australians in the country [26]. It is also home to the largest language group in Australia – Wiradjuri. The First Nations population in the study region ranges from 19% in Dubbo to 79% in Brewarrina [27]. The study began three months after COVID-19 was declared a global pandemic, and the Australian government began emphasizing the need for personal protective measures, including social distancing, hand sanitising and staying home as much as possible. At the time mask wearing had not been mandated and unlike other measures, the effectiveness of masks was being debated. No vaccine was available at this time.

### Participants

To be included in the study participants had to be aged 18 years and over, able to read and understand English and reside in Western NSW. Recruitment of all participants occurred between July and August 2020. Participants were recruited from two panel providers, Dynata and Qualtrics. Two were necessary as neither panel could provide sufficient respondents to meet our requirements in the region of interest. Panel data was used as it was a quick and efficient method of data collection and helped to overcome the problems of low response rates due to survey fatigue during the pandemic [28, 29]. In keeping with the non-probability nature of panel data, quota sampling was used to approximate a representative sample of participants [28, 30], with broad age and gender quotas imposed to reflect population statistics of the region. While the sample resembles the population there were some differences with females (63.4%) and those aged 18-29 (21.6%) overrepresented in the sample (Table 1).

**Table 1:** Survey respondent demographics

Sample characteristics compared with Population of the Western NSW Regions Surveyed			
Demographic characteristic	Sample (*n*=701)	Population of the Western NSW regions	Chi Square test
**Age brackets (Valid %)**			
18-29	21.6%	15.4%	
30-49	33.6%	31.5%	*p*=0.000
50-69	31.2%	33.5%	
70+	13.6%	19.5%	
**Gender**			
Female	436 (63.4%)	50.10%	*p*= 0.000
Male	249 (36.6%)	49.90%	
**Proportion of First Nations respondents**			
Aboriginal/TSI	60 (8.6%)	13%	*p*= 0.000
Non Aboriginal/TSI or Prefer not to say	641 (91.4%)	87%	

Due to the high number of First Nations people in this population, we engaged in additional recruitment through personal and community networks to improve the representation of First Nations participants. To facilitate access for First Nations Australians, information about the survey was shared through Elders groups, the project’s First Nations Reference Group, and people were given the opportunity to complete a paper-based version of the survey with the support of First Nations members of the research team (recognising the limited access to the internet in some regions)*.* As shown, in Table 1, the response rate for First Nations participants was 8.6%, this was not quite as high as the population in the region but was much higher than the 3.4% present in the NSW population [27].

### Survey

The online survey used in this study was an adaptation of a questionnaire developed for assessment of risk perceptions, anxiety, and behavioural responses of the general public during the early phase of the Influenza A (H1N1) pandemic [24]. The questionnaire fit the objectives of this study given the contextual similarities, theoretical models explaining health behaviour, design and reliability (Cronbach's alpha) of its constructs ranging from 0.6 to 0.9, with trends analysed over time [31]. A number of measures were put in place to ensure the validity of the measures. First, the language was adjusted slightly to fit the COVID context. Then the survey was examined and tested and refined using a focus group and with ongoing support from a specialist in survey design. To ensure the cultural safety and sensitivity of the measures a number of drafts were also examined by the First Nations Reference group. Once the survey was developed it was pilot tested with peers and each panel provider (Qualtrics and Dynata) ran a pilot test prior to deploying the instrument. Finally, the data was cleaned by the panel providers and again by the research team when the data sets were combined.

A number of variables were chosen because a holistic approach that includes individual demographics and cognitive, affective, social and cultural factors is recommended to assess risk perceptions [32]. Demographic measures included First Nations status, postcode, age, gender, education, rural or town/village location, proximity to medical services and living situation. Demographic measures were selected based on advice from the Project Advisory Group. This study analysed five items related to perceptions about COVID-19: perceived likelihood of contracting COVID-19 in the next 12 months, perceived harmfulness of the virus, how often people felt afraid, perception about respondents’ ability to do something about the virus and perceived economic impacts of the pandemic.

#### Data analysis

The data reported here come from a larger study. The same data collection methods were used in the larger study. This analysis is focused on those perceptions where there were significant differences between First Nations and non-First Nation respondents. Respondents from Murrumbidgee, Western and Far Western NSW Local Health Districts were identified by postcode. Respondents from other areas were excluded from the analysis.

Both descriptive and multivariate statistical analyses were used to assess links between First Nations status and other variables. Bivariate analyses using cross-tabulations were performed for the respondent’s First Nations status variable by anxiety about COVID-19 and socio-demographic characteristics, which were considered for multivariate analysis. Significant determinants were explored by Pearson’s chi-squared test and Fisher’s exact test. Multivariate logistic regression modelling for multivariable analyses was carried out to determine the influence of some covariates on the likelihood of experiencing anxiety for COVID-19, such as ‘feeling afraid’ and ‘perceiving harmful’ variables included in the survey.

The significant relationships between a variable and its effects were quantified by calculating the odds ratios with 95% confidence interval measures. The odds ratio (OR) in favour of ‘moderate’ and ‘severe’ forms of anxiety was computed for the selected group of covariates to suggest how many times the group of interest is more likely to belong to the target group compared to the reference group, i.e. ‘no’ anxiety. Two regression models were used separately for the two different anxiety-related response variables related to COVID-19 perceptions. The -2 Log Likelihood-based Chi-squared test was employed to check the statistical significance of the fitted model. Further details about these methods and analysis techniques are available in the existing literature [33]. IBM SPSS version 26 was utilized in all statistical analyses.

#### Ethics approval

Ethics approval was provided by the Aboriginal Health and Medical Research Council of NSW (AH&MRC) [1668/20] to enable the analysis of First Nations responses and the Charles Sturt University Human Research Ethics Committee (H20254).

## Results

There were 701 completed surveys collected. Paper-based surveys were completed by seven First Nations Australian respondents. The remaining 694 surveys were completed online. There were more female (*n*=447) than male respondents (*n*=254). Table 2 reports the survey respondents’ demographic characteristics. There were significant differences between First Nations (*n*=60) and non-First Nations (*n*= 639) respondents across all categories. First Nations respondents were significantly more likely to be female, to have a postgraduate degree, to live in a rural town more than 20 kilometres away from a health service and to be looking after children in their home compared to non-First Nations respondents.

**Table 2:** Background characteristics of the respondents and their percentage distribution by First Nations and non-First Nations status from selected study areas in Australia, 2021 (*n*=701)

Characteristic and categories	No. of participants^a^	%	First nations %	Non-First Nations or does not identify %	p-value
Sex					
Male	254	36.2	34.6	53.3	*p*=0.004
Female	447	63.8	55.4	46.7	
Age (in years)					
18-29	151	21.6	25.0	136	*p*=0.000
30-49	235	33.6	56.7	201	
50-69	218	31.2	13.3	210	
70 plus	95	13.6	5.0	92	
Education					
School up to Year 12	228	32.5	21.7	33.5	*p*=0.000
Trade/Diploma	257	36.7	28.3	37.4	
Bachelor	158	22.5	16.7	23.1	
Postgraduate	58	8.3	33.3	5.9	
Children under 18 at home					
Yes	224	75.2	92.9	72.3	*p*=0.003
No	74	24.8	7.1	27.7	
Residence					
Rural Town over 500 population	603	86.4	93.3	85.7	*p*=0.067
Rural Area	95	13.6	6.7	14.4	
Nearest health service					
Up to 20kms	632	90.2	78.3	91.3	*p*=0.01
More than 20kms	69	9.8	21.7	8.7	
Living situation					
Single person /couple without children	137	19.5	13.3	20.1	*p*=0.000
Couples/single parent with children	265	37.8	15	39.9	
Other household types	299	42.7	71.7	39.9	

^a^Total number of participants may differ due to missing data

Having compared the demographic characteristics of the First Nations respondents with non-First Nations members of the sample, in the next section, the groups are compared in terms of their perceptions about COVID-19. Table 3 includes five measures related to COVID-19 perceptions where there was a significant difference between the two groups: perceived likelihood of contracting COVID-19 in the next 12 months, perceived harmfulness of the virus, how often people felt afraid because of COVID-19, perceptions about respondents’ ability to do something about the virus and perceived economic impacts of the pandemic.

**Table 3:** COVID-19-related perceptions and anxiety characteristics of the participants and their percentage distribution by First Nations and non-First Nations status, 2021.

Factors	Number of observations (%)	First Nations	Non-First Nations	*p*-value
Likelihood of getting in next 12 months				
Not very likely	358 (52.7%)	53.90%	40.40%	*p*=0.000
Moderately likely	259 (38.1%)	40.50%	12.30%	
Very likely	62 (9.1%)	5.60%	47.40%	
Perceived Harmfulness				
Not very harmful	59 (8.4%)	8.80%	3.30%	*p*=0.028*
Moderately harmful	204 (29.1%)	30.20%	18.30%	
Very harmful	438 (62.5%)	61.10%	78.30%	
Feel Afraid				
Not often/Rarely	318 (45.4%)	47.00%	28.80%	*p*=0.022
Sometimes	209 (29.9%)	29.30%	35.60%	
Often/All of the time	173 (24.7%)	23.70%	35.60%	
There is nothing we can do about COVID-19				
Disagree	425 (60.7%)	63.4%	18.3%	
Neither agree nor disagree	169 (24.1%)	25.0%	21.7%	*p*=0.000
Agree	106 (15.1%)	11.6%	60.0%	
Economic impacts of COVID-19 not as bad as predicted				
Disagree	543 (77.7%)	80.8%	45.0%	
Neither agree nor disagree	89 (12.7%)	12.4%	16.7%	*p*=0.000
Agree	67 (9.6%)	6.9%	38.3%	

*p-value estimated using Fisher’s exact test

The results reflect a significantly higher level of anxiety among the First Nations group in the sample: they felt afraid more often, felt it was highly likely they would catch the virus and if they did catch the virus perceived that it would be very harmful.

- First Nations respondents (47.4%, *p*=0.000) were eight times more likely than non-First Nations respondents (5.6%, *p*=0.000) to indicate they were “very likely to get COVID-19 in the next 12 months.
- Although the majority of the sample considered the disease harmful, more First Nations respondents perceived COVID-19 to be very harmful (78.3% versus 61.1%, *p*=0.028), whereas non-First Nations respondents were more likely to consider the disease moderately harmful (30.2% versus 18.3%, *p*=0.028).
- Nearly half of non-First Nations respondents (47%, *p*=0.022) reported rarely feeling afraid about COVID-19 compared to only 28.8% of those who identified as First Nations.
- Two-thirds (63.6%, *p*=0.000) of First Nations respondents agreed that there was nothing they could do about COVID-19, whereas only 11.6% (*p*=0.000) of the rest of the sample agreed with this statement.
- In relation to the economic impacts of COVID-19, non-First Nations members of the sample (80.8%, *p*=0.000 ) were much more likely to expect a negative impact than First Nations respondents (45%, *p*=0.000).

The significant differences in other demographic characteristics included in Table 2 suggested that there may be more than First Nations status influencing the perceptions of each group, for example, education, age, gender, and population where people live and whether children were present in the household. The following section reports the results of two multinomial regression analyses examining the predictive relationship between the demographic characteristics of respondents and 1) how often they felt afraid of COVID-19 and 2) how harmful they perceived the virus to be.

In the first regression (Table 4), how often people reported feeling afraid about the virus was considered. Non-First Nations respondents were 60% less likely to report feeling afraid sometimes than First Nations respondents, illustrating a very different level of affect between these groups. Compared to those living in rural and remote areas, those living in towns were less likely to feel afraid sometimes (51.1%) and all of the time (79.6%). Males were six times more likely than females to report feeling afraid all of the time, and those with children under 18 in their household were 1.8 times more likely to report feeling afraid sometimes (when compared to those who did not feel afraid). Finally, those with a trade certificate or a diploma were 71.6% less likely to say they sometimes felt afraid than those with a postgraduate degree.

**Table 4:** Regression coefficients and odds ratios for the likelihood of moderate and severe forms of anxiety (i.e. feeling afraid) due to COVID-19 by some selected significant characteristics, including the First Nations status of the respondents^a^

Feel Afraid (Reference category is not afraid)						
	Afraid sometimes			Afraid often /all of the time		
Characteristic ^b^	B	Odds Ratio	95% CI	B	Odds Ratio	95% CI
Non-First Nations	-0.92	0.40***	(0.159, 1.006)	0.15	0.87	(0.124, 6.019)
Male	-0.05	0.95	(0.493, 1.825)	1.79	6.01**	(1.743, 20.721)
Children under 18	0.57	1.77*	(0.963, 3.238)	0.90	2.45	(0.605, 9.957)
Rural Town over 500ppl	-0.73	0.49*	(0.215, 1.070)	-1.60	0.20**	(0.050, 0.832)
Education						
Year 12	-0.86	0.42	(0.141, 1.274)	0.44	1.55	(0.117, 20.461)
Trade/Diploma	-1.26	0.28**	(0.096, 0.840)	0.17	1.18	(0.091, 15.345)
Bachelor	-0.59	0.55	(0.174, 1.752)	1.69	5.39	(0.456, 63.762)
**Model fitting information**						
	-2 Log Likelihood			135.208.		
	Chi-squared (df)			41.994 (14)****		

^a^Reference category of dependent variable is not afraid

^b^Omitted categories (i.e. reference class for each independent variable) not shown

*p<0·10; **p<0·05; ***p<0·01; ****p<0.001

In the second regression, the perceived harmfulness of the virus was examined (Table 5). First, Nations status was the only variable that was significantly related to perceptions of harmfulness. When compared to those who thought COVID-19 was not harmful, Non-First Nations respondents were 66% less likely than First Nations respondents to perceive COVID-19 as moderately harmful and 93% less likely than First Nations respondents to report that the virus was very harmful. Education level does not make a difference in this model.

**Table 5:** Regression coefficients and odds ratios for the likelihood of moderate and severe forms of anxiety (i.e. perceiving harmful) due to COVID-19 by some selected significant characteristics, including the First Nations status of the respondents^a^

Harmful (reference category is not harmful)						
	Moderately harmful			Very Harmful		
Characteristic ^b^	B	Odds Ratio	95% CI	B	Odds Ratio	95% CI
Non-First Nations	-1.16	0.31	(0.034, 2.899)	-2.73	0.07***	(0.008, 0.533)
Male	-0.56	0.57	(0.237, 1.365	-0.61	0.54	(0237, 1.240)
Children under 18	0.38	1.47	(0.619, 3.483)	0.02	1.02	(0.454, 2.279)
Rural Town over 500ppl	0.42	1.52	(0.458, 5.016	-0.42	0.66	(0.230, 1.881)
Education Trade/Diploma	0.23	1.26	(0.546, 2.903)	-0.032	0.97	(0.439, 2.130)
**Model Fitting information**						
	-2 Log Likelihood		113.622		.	
	Chi-squared (df)		28.588 (10)***			

^a^Reference category of dependent variable is not harmful

^b^Omitted categories (i.e. reference class for each independent variable) not shown

*p<0·10; **p<0·05; ***p<0·01; ****p<0.001

## Discussion

Overall, these findings reflect a much higher level of anxiety and fatalism amongst the First Nations respondents. There were some significant differences in risk perception and impacts of COVID-19 between First Nations and non-First Nations survey respondents and several demographic variables that predicted responses to COVID-19. First Nations respondents perceived COVID-19 to be more harmful than non-First Nations respondents to perceive a higher danger and vulnerability from the virus. . At the time the survey was conducted, there was no COVID-19 reported in Western NSW. Therefore, the finding of high perceptions of vulnerability from COVID-19 may reflect the media discourse about the high level of risk that First Nations people face in relation to COVID-19 and the potential impact on First Nations communities in other parts of Australia as well as confused messaging about risks to different places and sectors of the population [19, 21, 24].

There were significantly higher levels of anxiety among First Nations respondents (based on perceptions of fear and the harmfulness of the virus). Significantly higher levels of fatalism and a huge disparity in the perceived likelihood of catching the virus in the next 12 months point to the impact of COVID-19 anxiety on top of existing high levels of mental distress and social disadvantage [16]. All factors are consistent with a syndemic [10] indicating a much more serious impact of COVID-19 on First Nations Australians compared to non-First Nations Australians. First Nation’s survey respondents’ fears were justified because the Delta variant of COVID-19 quickly took hold in small communities with limited healthcare services, reflecting the pattern seen in other countries [16, 2]. Limitations in accessing healthcare services are also reflected in the results. Excluding rural populations from risk groups at the start of the pandemic was a dangerous oversight by the Australian government. The high proportion of First Nations people in Western NSW was not taken into account in vaccine rollout plans or the limited availability of healthcare services.

Other factors influencing perceptions of COVID-19’s harmfulness were also characteristic of many First Nations communities. Residing in a small rural town and living with children under the age of eighteen years were significantly predictive of concerns about COVID-19 harms. First Nations peoples typically live in larger extended family groups, including children, and in Western NSW, they are more likely to live in small rural communities than on farming properties [27, 34]. Frequently changing advice about the risks both to and from children directly impacts First Nations communities and likely heightened fear and concern about COVID-19 risks.

A high proportion of First Nations survey respondents had a post-school qualification (78% in the study compared to 42% of First Nations Australians nationally) [35]. However, most of the non-first Nations respondents only had school or vocational qualifications. High levels of education are likely to be consistent with good health literacy and better adherence to COVID-19 protective measures [15, 17, 19]. Lower levels of fear and perceptions of COVID-19 harmfulness among non-First-Nations respondents with lower education levels indicate a potentially blasé response to COVID-19 risks. Lower education levels are correlated with lower levels of concern about pandemic risks and typically result in less adherence to infection control measures, putting vulnerable groups at risk [36, 37, 17].

Education level is not the only issue at play in the effectiveness of health communications [18]. The levels of fear and perceptions of harmfulness found in the survey results should be expected when distrust of government and previous poor health care experiences are widespread for First Nations people [20]. There were no First Nations representatives in the daily government press conferences that delivered health advice even though there were frequent mentions of risks to ‘the regions’ [16, 20]. Heterogeneity of the population means diverse capacity to understand and apply pandemic related information [19]. Health messages could better target their intended audience by directly addressing the specific concerns and healthcare experiences of distinct populations such as First Nations communities in rural areas, via co-design of health communications and dissemination strategies [9]. The Aboriginal Community Controlled health sector worked hard to inform First Nations communities about COVID-19 risks, including closing remote Northern Territory communities and developing localised social media campaigns for these sites where subsequently no COVID-19 cases were reported during 2020 [21].This approach was not applied nation-wide. As the pandemic continues, the development of specific health communications for rural people in NSW is required with tailored options for First Nations Australian communities in the region.

### Strengths and Limitations

There has not been a First Nation’s informed investigation of First Nations Australian’s perceptions of COVID-19 in Western NSW. The results suggest key areas for more nuanced health communications to address. However, the small sample size (*n*=60) was not representative of the First Nations population in Western NSW (8.4% in the sample vs 13% in the region), and the results do not represent the experiences or perceptions of First Nations Australians in other areas. However, the proportion of First Nations respondents in this study is greater than the population proportion nationally and more than reported in other Australian studies [23, 24]. Furthermore, the study included 78% of First Nations respondents with a post-school qualification compared to 42% nationally [35]. This result suggests, however, that education is less likely to be a factor in COVID-19-related anxiety than other factors, such as mental distress and poor experiences of healthcare and government interventions [20, 10].

## Conclusion

Health communications for First Nations Australians in rural areas should be designed and delivered by First Nation’s Australians from those areas because they understand the rural context in which people live. This principle of First Nations-led design is critical to all health policy and planning. The Australian Government should include rural areas in planning pandemic responses, recognising that First Nations populations are a significant proportion of the rural population with different risk factors and concerns than those of non-First Nations peoples, creating syndemic conditions.

## Supplementary Information

**Additional file 1.**

## Data Availability

The datasets generated during and/or analysed during the current study are available from the corresponding author on reasonable request.
