# Supplementary material for: A comparison of rural Australian First Nations and Non-First Nations survey responses to COVID-19 risks and impacts: implications for health communications
Source: BMC Public Health. 2022 Jun 30;22:1276. doi: 10.1186/s12889-022-13643-6 (PMC9245509; doi:10.1186/s12889-022-13643-6)
Supplement: Supplementary file 1 — Additional file 1. [file 12889_2022_13643_MOESM1_ESM.docx]

**Understanding the impact of COVID-19 on perceptions, health beliefs, protection motivations and the impact of those on prevention measures in rural and remote western NSW during the outbreak.**

# Survey Measures

**Note: * = A new variable created**

**Demographics:**

- - Gender
  - Age Bracket* (4 Categories)
  - First Nationsity* (Y/N, Prefer not to say)
  - Education level (7 Categories)
  - Living Situation (7 Categories)
  - Remoteness (5 Categories)
  - Proximity2MedicalServices* (Score 1-6; 1=close)
  - Employment (3 test items)
  - Children living at home (Y/N)
  - Pre-existing health condition (Y/N)
  - Physical Wellbeing (Score 1-5; 1=Very poor)
  - Mental/Emotional Wellbeing (Score 1-5; 1=Very poor)

**COVID-19 (knowledge and risk perception)**

- KnowledgeScore_6* (Score 0-6; 6=High knowledge level)
- Seriousness of the virus (Score 1-5; 5=Extremely serious)
- Likelihood of getting virus (Score 1-5; 5=Extremely likely)
- Harmfulness of virus (Score 1-5; 5=Extremely harmful)
- Frequency of thinking/worry/fear (Frequency_Worry*) (Score 1-5; 5=All the time)
- COVID-19 symptoms in the first 2 months (Y/N)
  - - Sought medical advice (Y/N)

**Preventative measures**

- Effectiveness_PrevMeasures* (Score 1-5; 5=Very effective)
- Ease_Compliance* (Score 1-4; 4=Very Easy)
- Actual_Compliance* (Score 1-5; 5=All the time)
- Intention to comply ( Score 1-5; 5=completely)
- OtherProtectiveMeasures* (Y/N)

**Agreement with Impact Statements**

- - Media exaggeration (Score 1-5; 5=Strongly agree)
  - Government exaggeration (Score 1-5; 5=Strongly agree)
  - Health impacts not as bad as predicted (Score 1-5; 5=Strongly agree)
  - Economic impacts not as bad as predicted (Score 1-5; 5=Strongly agree)
  - Nothing can be done (Score 1-5; 5=Strongly agree)
  - Just have to accept it (Score 1-5; 5=Strongly agree)
  - I will move (Score 1-5; 5=Strongly agree)
  - I will stay indoors (Score 1-5; 5=Strongly agree)

**Information-seeking behaviour**

- COVID-19 (Y/N)
- Rules (Y/N)
- Preventative Measures (Y/N)
- InformationSeekingBehavior* (Score 1-3; 3=high frequency)

**Information Sources**

Frequency of use

- Television (e.g., News channels, programs) (Score1-5; 5=All the time)
- Radio (Score1-5; 5=All the time)
- Newspapers (Online/Offline) (Score1-5; 5=All the time)
- Friends / Family / Peers (Score1-5; 5=All the time)
- Local GP / Local Pharmacist (Score1-5; 5=All the time)
- Medical Centre/Hospital (Score 1-5; 5=all the time)
- Influential community member / leader (Score1-5; 5=All the time)
- Government websites (Score1-5; 5=All the time)
- Government press conferences (Score1-5; 5=All the time)
- World Health Organisation (Score1-5; 5=All the time)
- Social media (e.g., Twitter, Facebook, Instagram, YouTube) (Score1-5; 5=all the time)
- Influential social media personality / leader (Score1-5; 5=All the time)
- Google / Bing / Yahoo (Score1-5; 5=All the time)

Quantum of influence

- Television (e.g., News channels, programs) (Score1-5; 5=A great influence)
- Radio (Score1-5; 5=A great influence)
- Newspapers (Online/Offline) (Score1-5; 5=A great influence)
- Friends / Family / Peers (Score1-5; 5=A great influence)
- Local GP / Local Pharmacist (Score1-5; 5=A great influence)
- Medical Centre/Hospital (Score 1-5; 5=A great influence)
- Influential community member / leader (Score1-5; 5=A great influence)
- Government websites (Score1-5; 5=A great influence)
- Government press conferences (Score1-5; 5=A great influence)
- World Health Organisation (Score1-5; 5=A great influence)
- Social media (e.g., Twitter, Facebook, Instagram, YouTube) (Score1-5; 5=A great influence)
- Influential social media personality / leader (Score1-5; 5=A great influence)
- Google / Bing / Yahoo (Score1-5; 5=A great influence)

Quantum of influence of types of information sources

All: (Score1-5; 5=A great influence)

- Influence_Mass Media*: TV, Newspapers, Radio
- Influence _Interpersonal Channels*: Friends / Family / Peers / Local GP / Local Pharmacist / Influential community member / leader
- Influence _Social Media*: Social media / Influential social media personality
- Influence _Official Sources*: Government websites / Government press conferences / WHO / Medical Centre / Hospital
- Influence _Internet Search Engines*: Google / Bing / Yahoo

**Most Trusted Information Source**

Single Categorical selection:

- Television (e.g., News channels, programs)
- Radio
- Newspapers (Online/Offline)
- Friends / Family / Peers
- Local GP / Local Pharmacist
- Medical Centre/Hospital
- Influential community member / leader
- Government websites
- Government press conferences
- World Health Organisation
- Social media (e.g., Twitter, Facebook, Instagram, YouTube))
- Influential social media personality/leader
- Google / Bing / Yahoo

**Missing Information**

- Was there Information you couldn’t find (Y/N)
